# Association between antimicrobial usage and resistance on commercial broiler and layer farms in Bangladesh

**DOI:** 10.3389/fvets.2024.1435111

**Published:** 2024-08-29

**Authors:** Mohammad Foysal, Tasneem Imam, Shetu B. Das, Justine S. Gibson, Rashed Mahmud, Suman D. Gupta, Guillaume Fournié, Md. Ahasanul Hoque, Joerg Henning

**Affiliations:** ^1^Chattogram Veterinary and Animal Sciences University, Chattogram, Bangladesh; ^2^School of Veterinary Science, The University of Queensland, Gatton, QLD, Australia; ^3^Gulbali Institute, Charles Sturt University, Wagga Wagga, NSW, Australia; ^4^School of Agricultural, Environmental and Veterinary Sciences, Charles Sturt University, Wagga Wagga, NSW, Australia; ^5^Royal Veterinary College, University of London, London, United Kingdom; ^6^INRAE, VetAgro Sup, UMR EPIA, Université de Lyon, Marcy l’Etoile, France

**Keywords:** *Escherichia coli*, *Salmonella* spp., broiler, layer, commercial farm, Chattogram

## Abstract

Antimicrobial resistance has emerged as a significant health problem worldwide, including in Bangladesh, where chickens are an important protein source for human nutrition. One of the factors accelerating the development of antimicrobial resistance is the inappropriate use of antimicrobials on commercial chicken farms. A cross-sectional study was conducted in 2019 on 140 commercial chicken farms in the Chattogram district of Bangladesh to investigate the association between antimicrobial use and resistance in *Escherichia coli* and *Salmonella* spp. cultured from cloacal swabs of chickens and from the poultry shed environment. All *E. coli* and *Salmonella* spp. isolates were resistant to multiple antimicrobial classes, including those categorized as “Highest Priority Critically Important Antimicrobials” for human medicine. Notably, resistance was observed in *E. coli* isolates from farms that did not use these antimicrobial classes in the current production cycle. For example, although quinolones were not used on 43.9% of *E. coli* positive farms, 95.7% of these farms had quinolone-resistant *E. coli* isolates. The results of the path analysis revealed that there was a “direct effect” of the frequency of antimicrobial usage on “high” resistance, with resistance increasing when antimicrobials were administered more frequently (*β* = 0.28, *p* = 0.002). There was a “direct effect” of the purpose of antimicrobial use on “low” resistance, with resistance marginally decreasing when antimicrobials were administered solely for therapeutic use (*β* = −0.17, *p* = 0.062), but increasing when they were used prophylactically. Overall, the study results could be used to educate farmers on better practices for antimicrobial administration, and to guide government agencies to update policies on antimicrobial use and resistance surveillance in the poultry sector of Bangladesh.

## 1 Introduction

Antimicrobial resistance in humans has been associated with the administration of antimicrobials in livestock (1). Antimicrobial resistant bacteria or their resistance genes can be transmitted to humans through direct contact with animals, consumption of animal products, or through environmental exposure (2).

In Bangladesh, two poultry production systems are established: backyard and commercial farming (3). Backyard farmers typically raise an average of seven birds (4), and commercial producers up to 20,000 birds (5). Commercial poultry farmers in Bangladesh predominately use antimicrobials to prevent and treat poultry diseases, but they are often misused (6). The lack of proper monitoring of antimicrobial usage and inadequate awareness regarding the impact of antimicrobial use on antimicrobial resistance emergence (7), the influence of feed and chick traders on husbandry practices (8), low biosecurity (9), financial constraints (10), and limited access to veterinary services (11) promote the misuse of antimicrobials, which may lead to the development of resistance (1). In Bangladesh, beta-lactams, tetracyclines, sulphonamides, fluoroquinolones, polymyxins, and aminoglycosides are frequently used in poultry to treat and prevent disease (12–14). For example, in Bangladeshi broiler farms, polymyxin (30.14%) was the most used antimicrobial, followed by sulphonamides (26%), tetracyclines (20.6%), quinolones (19.2%) (12). On layer farms, quinolones (1.66–22.5%), beta lactams (16.66%), tetracyclines (10.83%), and sulphonamides (3.33%) were commonly used antimicrobials (13). Resistance to these antimicrobials have been found in *Escherichia coli* and *Salmonella* spp. cultured from commercial chickens (12–14). However, these studies have either evaluated antimicrobial use or resistance, but not both together.

Under the “One Health” paradigm, the World Organization for Animal Health (WOAH), the Food and Agriculture Organization (FAO) and the World Health Organization (WHO) have provided recommendations to reduce the global use of antimicrobials and limit the risk of global spread of resistant bacteria (15). A database monitoring the type and quantities of antimicrobial agents used in animals was launched in October 2015 (15). The FAO has also launched an Action Plan to minimize the impact of antimicrobial resistance (16). This plan aims to develop the capacity for monitoring the use of antimicrobials in food animals (16), while WHO has developed a Global Action Plan to reduce the non-judicious use of antimicrobials in animals and humans (17).

The Bangladesh government formulated a National Action Plan (2017–2022) to tackle antimicrobial resistance in humans, livestock and fisheries and strengthen infectious disease prevention and control (18). However, for implementation, reliable data on antimicrobial use and resistance in the livestock sector is required. Our previous research assessed antimicrobial usage on commercial chicken farms, identifying that “Medically Important Antimicrobials” were frequently purchased from feed and chick traders, and often used in the absence of clinical signs and without adherence to withholding periods (6).

*Escherichia coli* is a commensal bacterium in food producing animals and is commonly used to monitor the development of antimicrobial resistance and the risk of antimicrobial resistant bacteria transmission from animals to human (19). Colibacillosis, caused by avian pathogenic *E. coli* (APEC), is among the most prevalent bacterial infections in chickens and other poultry in Bangladesh (20). Chickens are continually exposed to *E. coli* through feces, water, dust, and their environment (20). Since *E. coli* are always present in the gastrointestinal tract of birds, they easily spread via feces (20). In a study conducted on a Bangladeshi chicken farm, 36 of 99 samples, comprising internal organs, feces, and air from inside poultry sheds, tested positive for the APEC-associated virulence genes *fimC*, *iucD*, and *papC* (21). *Salmonella* is a zoonotic foodborne bacterium and one of the major causes of human food borne disease (22). Both bacteria, which are often cultured from healthy animals at slaughter, are used worldwide as bioindicators for antimicrobial susceptibility.

In this study, we aimed to quantify the association between antimicrobial usage and susceptibility of *E. coli* and *Salmonella* spp. cultured from samples collected on commercial chicken farms in Bangladesh. In addition, we aimed to identify farm management factors associated with the increased antimicrobial resistance.

## 2 Materials and methods

### 2.1 Field data collection

**Figure 1:**
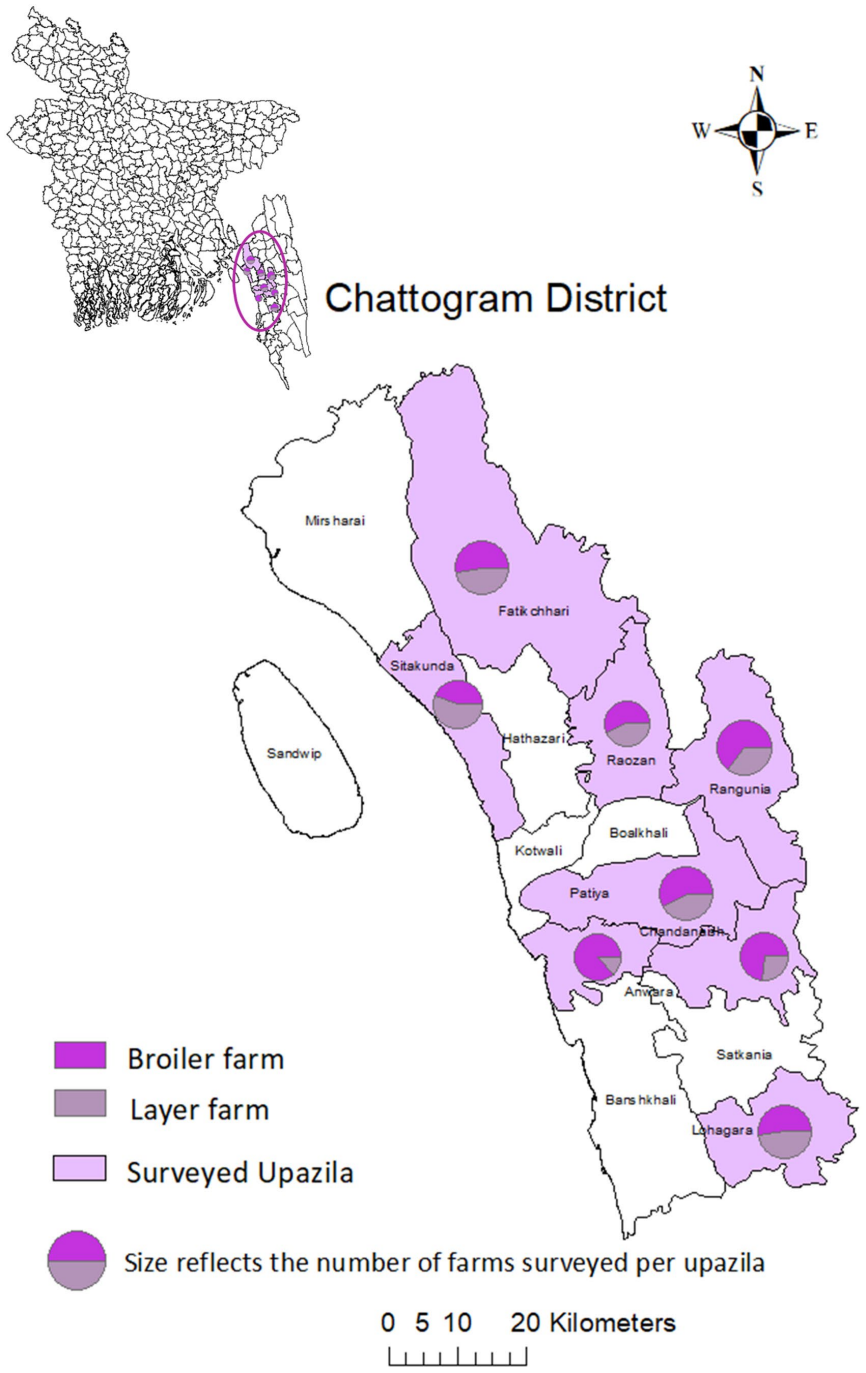
Commercial layer and broiler farms in the Chattogram district of Bangladesh. The eight *upazilas* sampled are highlighted in purple. Pie charts display the number of farms by farm type (broiler farm, layer farm), with the size of the pie charts representing the number of farms sampled per *upazila*.

A cross-sectional study was conducted from February to May 2019 on 140 commercial layer and broiler farms from eight *upazilas* (subdistricts) in the Chattogram district of Bangladesh (Figure 1).

Data collection included sampling chickens and their environment and the administration of a structured questionnaire to obtain data on antimicrobial usage, and farm management practices implemented on these farms, as described previously (6). Briefly, commercial chicken farms were chosen from a sampling frame of 1748 commercial chicken farms using simple random sampling by applying the syntax RANDBETWEEN in Microsoft Excel (Microsoft Corporation, 2010). The number of farms per *upazila* was maintained using probability proportional to size sampling.

On each farm, the following strategy was followed to select the chicken shed of interest:

- If the same number of antimicrobials was used in all sheds, the shed with the oldest chickens was selected.
- If the number of antimicrobials used across sheds differed, then the shed with the highest number of antimicrobials used was selected.

The structured questionnaire was validated by piloting it on five layer and five broiler farms. Additionally, photographs of antimicrobial packages were taken to cross-check the information provided. If available, photographs of the drug registration book kept on the farms were also taken to verify the data further.

Both cloacal and environmental swab samples were collected from the selected shed using the following strategy:

- Cloacal swab samples were collected from five randomly selected healthy chickens and pooled into a 15 mL sterile falcon tube.
- Environmental swab samples were collected from the four corners and the middle of the chicken shed and pooled into a falcon tube containing buffered peptone water (BPW) (Neogen Corporation, 620 Lesher Place, Lansing MI 48912, United States).

Samples were kept in an insulated box containing ice packs and transported to the Poultry Research and Training Center (PRTC) laboratory in Chattogram, Bangladesh within 4–6 h. The samples were stored at −20°C until analysis. Bacterial culture and antimicrobial susceptibility testing were conducted between June and September 2019.

Only farmers actively engaged in the management of poultry farming were interviewed. One farmer from each farm was interviewed. Verbal consent was obtained from the farmers for the interview and collection of cloacal and environmental samples. Interviews were conducted by trained researchers from the Chattogram Veterinary and Animal Sciences University (CVASU).

### 2.2 Bacterial culture

*Salmonella* and *E. coli* were isolated by standard microbiological methods according to ISO 6579-1: 2017 and ISO 7251: 2005, respectively (Neogen, Lansing MI) (23).

All positive isolates were stored at −80°C in brain heart infusion broth with 50% glycerol (Neogen Corporation) (24). Identification was confirmed by Vitek (VITEK IVP, Inc., United States) at the Bangladesh Livestock Research Institute (BLRI). Details of the bacterial culture are summarized in the Supplementary material.

### 2.3 Antimicrobial susceptibility testing of *Escherichia coli* and *Salmonella* spp.

All *E. coli* and *Salmonella* spp. underwent Kirby-Bauer disk diffusion antimicrobial susceptibility testing against 12 antimicrobials following the Clinical and Laboratory Standard Institute (CLSI) guidelines (25). Details of antimicrobial susceptibility testing are summarized in the Supplementary material.

These antimicrobials were selected because they were identified as being commonly used on chicken farms in Bangladesh (12, 26). As resistance for one antimicrobial in a class often selects for resistance for other antimicrobials in that class (27), we categorized the antimicrobials into classes for data analysis. If any antimicrobial in the class was resistant, the bacteria was classified as resistant to that antimicrobial class.

### 2.4 Data analysis

A farm was classified as positive for *E. coli* or *Salmonella* spp. if either a cloacal or environmental sample tested positive for the bacteria.

*E. coli* and *Salmonella* spp. isolates identified as “resistant” or “intermediate” in the susceptibility testing were categorized as “resistant” for further data analysis. When a farm tested positive for *E. coli* or *Salmonella* spp., the cloacal or environmental sample with resistance to the largest number of antimicrobial classes determined the farms’ level of resistance. The association between usage and non-usage of antimicrobial classes and, resistance and susceptibility to these antimicrobial classes was explored using scatter plots and the Fisher’s exact test (28).

Multiclass resistance was defined as “resistance to antimicrobials of three or more different classes” (29).

Path analysis was used to examine potential relationships either in a “direct” or “indirect” way among a set of observed variables (30). A “direct” relationship exists when an exogenous variable (in regression, known as an independent variable) directly affects an endogenous variable (known as an outcome variable), while an “indirect” relationship occurs when an exogenous variable affects an endogenous variable through a mediator variable (30). The “indirect” relationship can involve one or more mediator variables between the exogenous variable and the endogenous variable (31). Path coefficients (*β*) are then estimated to quantify the relationships between variables along a hypothesized pathway (30).

We utilized path analysis to explore the relationship between published management factors related to antimicrobial usage that could directly or indirectly increase antimicrobial resistance in *E. coli*. Details on the management factors that could potentially increase antimicrobial usage in *E. coli* on commercial chicken farms are shown in Table 1. Path analysis was not conducted for *Salmonella* spp. because this bacterium was cultured from only a limited number of farms.

**Figure 2:**
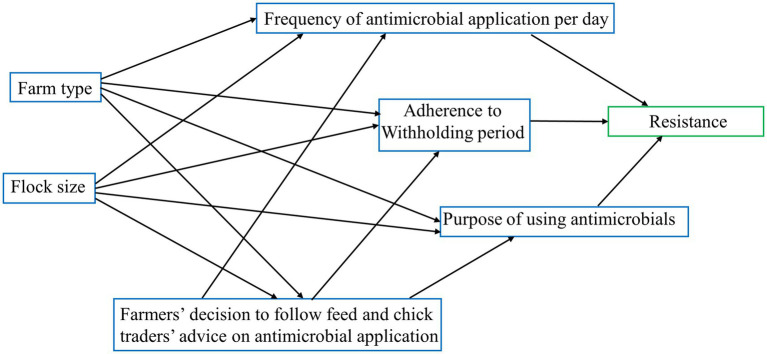
Hypothesized pathway model of commercial chicken farm management practices that potentially could have an effect on increasing resistance to antimicrobial classes used on these farms.

A diagram showing the hypothesized direct and indirect farm management pathways that could impact antimicrobial resistance on these farms is shown in Figure 2. For the path analysis, we classified resistance into two categories: “high” resistance to more than 5 antimicrobial classes (coded as “1”) and “low” resistance to up to 5 antimicrobial classes (coded as “0”).

**Table 1:** Management factors related to antimicrobial usage that potentially could have an effect on increasing resistance in *E. coli* on commercial chicken farms.

Predictors	Categories	Results
Farm type	Layer	Layer farms have been shown to have a higher prevalence of antimicrobial resistance (11).
	Broiler	
Flock size	≤2,500 birds	Larger flock sizes have been associated with a higher prevalence of antimicrobial resistance (11).
	>2,500	
Farmers’ decision to follow advice provided by feed and chick traders on the administration of antimicrobials	Not or rarely followed	Farmers who purchased antimicrobials from feed and chick traders were more likely to misuse antimicrobials (8) and it is hypothesized that these farms may have a higher prevalence of antimicrobial resistance.
	Frequently or always followed	
Purpose of using antimicrobials	Prophylactic with or without therapeutic	Prophylactic administration of antimicrobials contributes to a higher prevalence of antimicrobial resistance (32).
	Only therapeutic	
Frequency of antimicrobial administration per day	Once	Frequent administration of antimicrobials contributes to a higher prevalence of antimicrobial resistance (33).
	Twice	
	Three times	
Adherence to the withholding period	No	Non-adherence to withdrawal periods might be associated with higher prevalence of antimicrobial resistance (34).
	Yes	

Variables with no significant “direct” and/or “indirect” effect at p < 0.05 on increasing the level of antimicrobial resistance were not included in the final path model. The fit of the path model was assessed using the Chi-square (*χ*)^2^ statistic, with a *p*-value >0.05 indicating a good fit (35), the Root Mean Square Error of Approximations (RMSEA), with a value <0.05 indicating a good fit and a value up to 0.08 indicating an acceptable fit (36), the Comparative Fit Index (CFI), with a value >0.95 indicating very good fit and ≥ 0.90 an acceptable fit (35), and the Standard Root Mean Square Residuals (SRMR) with a value ≤0.05 indicating a close-fitting model and a value between 0.05 up to 0.10 an acceptable fit (36).

Descriptive analysis was conducted using STATA 16 (StataCorp®, 2019). The Stata 16.0 SEM Builder was used for the path analysis (STATA 16, Statacorp®, 2019). R 4.0.3 was used to create the scatterplot (“ggplot2,” R Core Team®, 2020). The map was created using ArcGIS version 10.8.1 (Redlands, CA: Environmental Systems Research Institute, Inc., 2011).

## 3 Results

From the 137 commercial farms where antimicrobials were used during the current production cycle, *E. coli* were cultured from 107 (78.1%) farms, and *Salmonella* spp. from 11 (8.0%) farms. From 107 farms, 79 (73.8%) had *E. coli* cultured exclusively from cloacal swabs, 81 (75.7%) from environmental swabs, and 53 (49.5%) from both swabs. From 11 farms, 3 (27.3%) had *Salmonella* spp. cultured exclusively from cloacal swabs, 10 (90.9%) from environmental swabs, and 2 (18.2%) from both swabs.

### 3.1 Antimicrobial usage

On *E. coli* positive farms, the most used antimicrobial classes (either alone or in combination with other antimicrobials) in the current production cycle were quinolones (56.1%, 60/107), tetracyclines (50.5%, 54/107), and polymyxins (43.0%, 46/107). On *Salmonella* spp. positive farms, tetracyclines (63.6%, 7/11), polymyxins (63.6%, 7/11), and beta lactams (54.5%, 5/11) were most used. On both *E. coli* and *Salmonella* positive farms, polymyxins and quinolones were mostly used (55.6%, 5/9), followed by beta lactams (44.4%, 4/9), macrolides (33.3%, 3/9) and sulphonamides (33.3%, 3/9).

### 3.2 Antimicrobial resistance

On an isolate level, all *E. coli* isolates collected from cloacal samples were resistant to beta lactams and tetracyclines, followed by macrolides (98.7%, 78/79), sulphonamides (97.5%, 77/79), quinolones (92.4%, 73/79), aminoglycosides (82.3%, 65/79) and polymyxins (3.8%, 3/79). All *E. coli* isolates collected from environmental samples were resistant to beta lactams and tetracyclines, followed by macrolides (98.8%, 80/81), sulphonamides (95.1%, 77/81), quinolones (86.4%, 70/81), aminoglycosides (74.0%, 60/81) and polymyxins (3.7%, 3/81) (Table 2). All *E. coli* isolates resistant to antimicrobials are presented in Supplementary Table S1.

**Table 2:** Results of antimicrobial susceptibility testing of *E. coli* and *Salmonella* spp. isolates cultured from cloacal and environmental samples collected on commercial chicken farms in the Chattogram district of Bangladesh.

Antimicrobial classes	*E. coli* resistance % (*N* farms)			*Salmonella* resistance % (N farms)		
	Overall (*N* = 107)	Cloacal (*N* = 79)	Environmental (*N* = 81)	Overall (*N* = 11)	Cloacal (*N* = 3)	Environmental (*N* = 10)
Beta lactams	100.0 (107)	100.0 (79)	100.0 (81)	100.0 (11)	100.0 (3)	100.0 (10)
Tetracyclines	100.0 (107)	100.0 (79)	100.0 (81)	90.9 (10)	100.0 (3)	90.0 (9)
Quinolones	95.3 (102)	92.4 (73)	86.4 (70)	100.0 (11)	100.0 (3)	100.0 (10)
Macrolides	100.0 (107)	98.7% (78)	98.8 (80)	Not performed		
Sulphonamides	100.0 (107)	97.5 (77)	95.1 (77)	54.5 (6)	100.0 (3)	30.0 (3)
Aminoglycosides	86.0 (92)	82.3 (65)	74.0 (60)	81.8 (9)	33.3 (1)	80.0 (8)
Polymyxins	5.6 (6)	3.8 (3)	3.7 (3)	18.2 (2)	0.0 (0)	20.0 (2)

Resistance includes isolates identified with resistant and intermediate susceptibility.

All *Salmonella* spp. isolates collected from cloacal samples were resistant to beta lactams, tetracyclines, quinolones, and sulphonamides followed by aminoglycosides (33.3%, 1/3). All *Salmonella* spp. isolates collected from environmental samples were resistant to beta lactams and quinolones, followed by tetracyclines (90.0%, 9/10), aminoglycosides (80.0%, 8/10), sulphonamides (30.0%, 3/10) and polymyxins (20.0%, 2/10) (Table 2). All *Salmonella* spp. isolates resistant to antimicrobials are presented in Supplementary Table S1.

On a farm level, all *E. coli* positive farms (*N* = 107) were resistant to beta lactams, tetracyclines, macrolides and sulphonamides, followed by quinolones (95.3%, 102/107), and aminoglycosides (86.0%, 92/107). A relatively low resistance was found for polymyxins (5.6%, 6/107). All *Salmonella* positive farms (*N* = 11) were resistant to beta lactams and quinolones, followed by tetracyclines (90.9%, 10/11), aminoglycosides (81.8%, 9/11) and sulphonamides (54.5%, 6/11); while low resistance was identified for polymyxins (18.2%, 2/11) (Table 2).

Only 3.7% (4/107) of the *E. coli* positive farms were resistant to four antimicrobial classes, 15.9% (17/107) were resistant to five, 74.8% (80/107) to six and, 5.6% (6/107) were resistant to all seven antimicrobial classes. Alternatively, 18.2% (2/11) of the *Salmonella* spp. positive farms were resistant to three antimicrobial classes, 27.3% (3/11) were resistant to four, 36.4% (4/11) to five, and 18.2% (2/11) were resistant to six antimicrobial classes. All *E. coli* and *Salmonella* spp. isolated were multiclass resistant.

### 3.3 Association between antimicrobial usage and antimicrobial resistance

**Figure 3:**
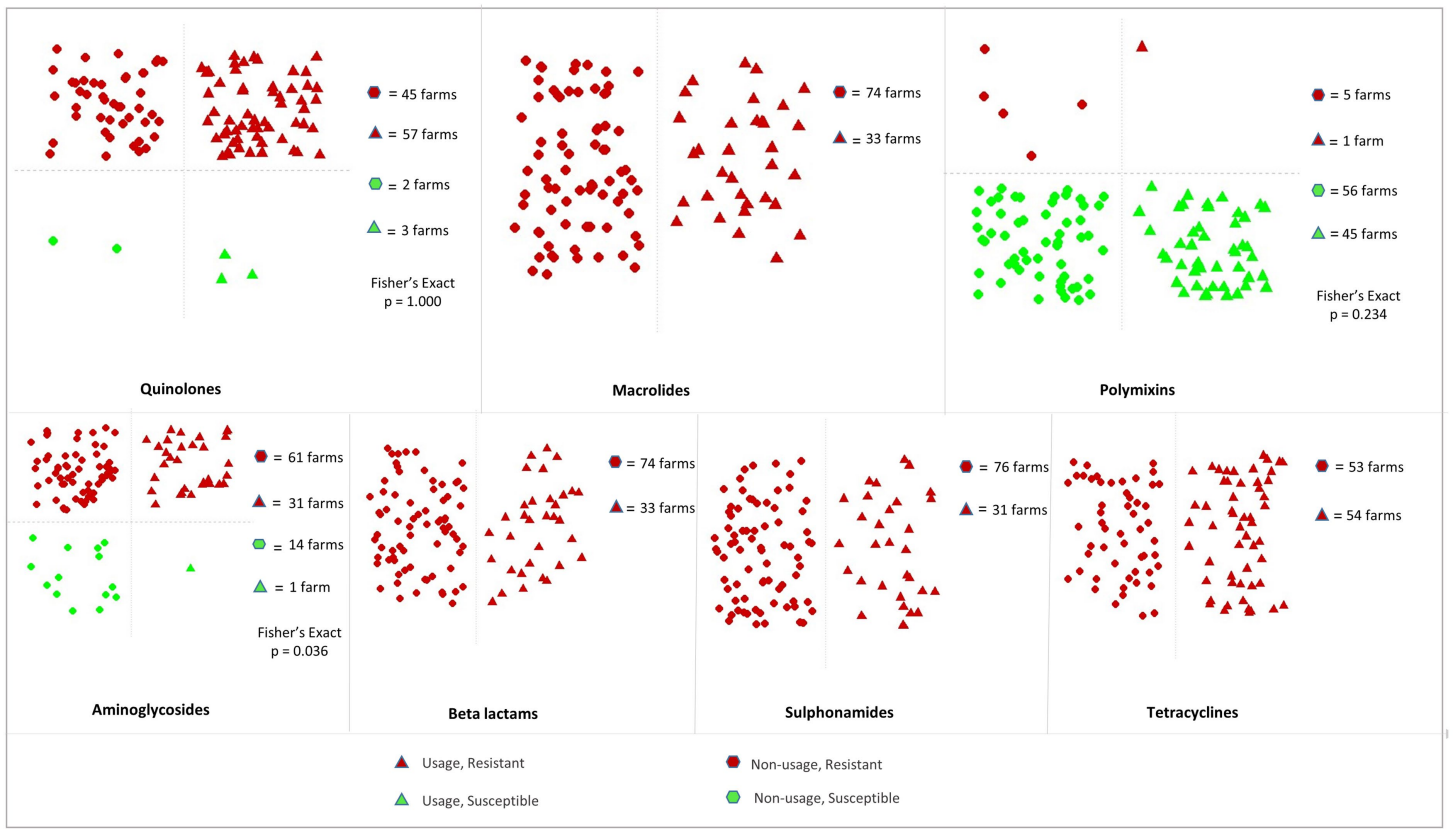
Usage and resistance pattern of antimicrobial classes on commercial chicken farms in the Chattogram district of Bangladesh where *E. coli* was cultured. Each data point represents a *E. coli* positive farm. The data points are jittered to avoid overlapping.

Supplementary Table S2 shows the frequencies of farms using antimicrobial classes on *E. coli*-positive and *Salmonella* spp.-positive commercial poultry farms, while the frequency of *E. coli*-positive and resistant farms where antimicrobials classes were not used in the current production cycle are presented in Supplementary Table S3. The relationship of usage and resistance pattern of quinolones, macrolides, polymyxins, aminoglycosides, beta lactams, sulphonamides, and tetracyclines is presented in Figure 3.

Our results highlight that *E. coli*-positive farms were frequently resistant to antimicrobial classes even when these classes were not reported to have been used during the studied production cycle. Indeed, quinolones, classified among the “Highest Priority Critically Important Antimicrobials” (HPCIAs) (37) were not reported being used on 43.9% (47/107) of the farms where *E. coli* was cultured, but 95.7% (45/47) of these non-user farms had quinolone-resistant *E. coli* isolates. Similarly, all isolates on *E. coli*-positive farms (100%, 74/74) were resistant to macrolides, beta lactams, sulphonamides and tetracyclines, despite the reported usage of these antimicrobial classes ranging between 29.0–50.5%. In contrast, polymyxins (colistin) were not used on 57.0% (61/107) of *E. coli*-positive farms, and only 8.2% (5/61) of these farms had polymyxin-resistant *E. coli* cultured. The frequency of polymyxin-resistance on farms that used polymyxins did not differ significantly from those farms that did not use polymyxins (*p* = 0.234). However, aminoglycosides were not used on 70.1% (75/107) of *E. coli* positive farms, but 81.3% (61/75) of these non-user farms had aminoglycosides-resistant *E. coli* isolates (*p* = 0.036).

### 3.4 Association between farm management factors and antimicrobial resistance

Descriptive statistics of management procedures used on commercial chicken farms that were considered as predictors in the path analysis are summarized in Table 3.

**Table 3:** Descriptive statistics of the management factors related to antimicrobial usage which potentially have an effect on increasing resistance in *E. coli* on commercial chicken farms.

Management factors across the surveyed farms (*N* = 107)		% (*N*)
Farm type	Layer	42.1 (45)
	Broiler	57.9 (62)
Flock size	≤2,500 birds	77.6 (83)
	>2,500	22.4 (24)
Farmers’ decision to follow advice provided by feed and chick traders on the administration of antimicrobials	Not or rarely followed	72.0 (77)
	Frequently or always followed	28.0 (30)
Purpose of using antimicrobials	Prophylactic with or without therapeutic use	86.0 (92)
	Only therapeutic use	14.0 (15)
Frequency of antimicrobial administration per day	Once	5.6 (6)
	Twice	28.0 (30)
	Three times	66.4 (71)
Adherence to the withholding period	No	56.1 (60)
	Yes	43.9 (47)

Out of the 107 *E. coli* positive farms, 72.3% (60/83) of farmers rearing small flocks (≤2,500 birds) used antimicrobials three times per day, compared to 45.8% (11/24) of farmers with larger flocks (>2,500 birds) (*p* = 0.003). The proportion of farmers using antimicrobials therapeutically only was higher among those rearing large flocks (29.2%, 7/24) than those rearing small flocks (9.6%, 8/83) (*p* = 0.023).

**Figure 4:**
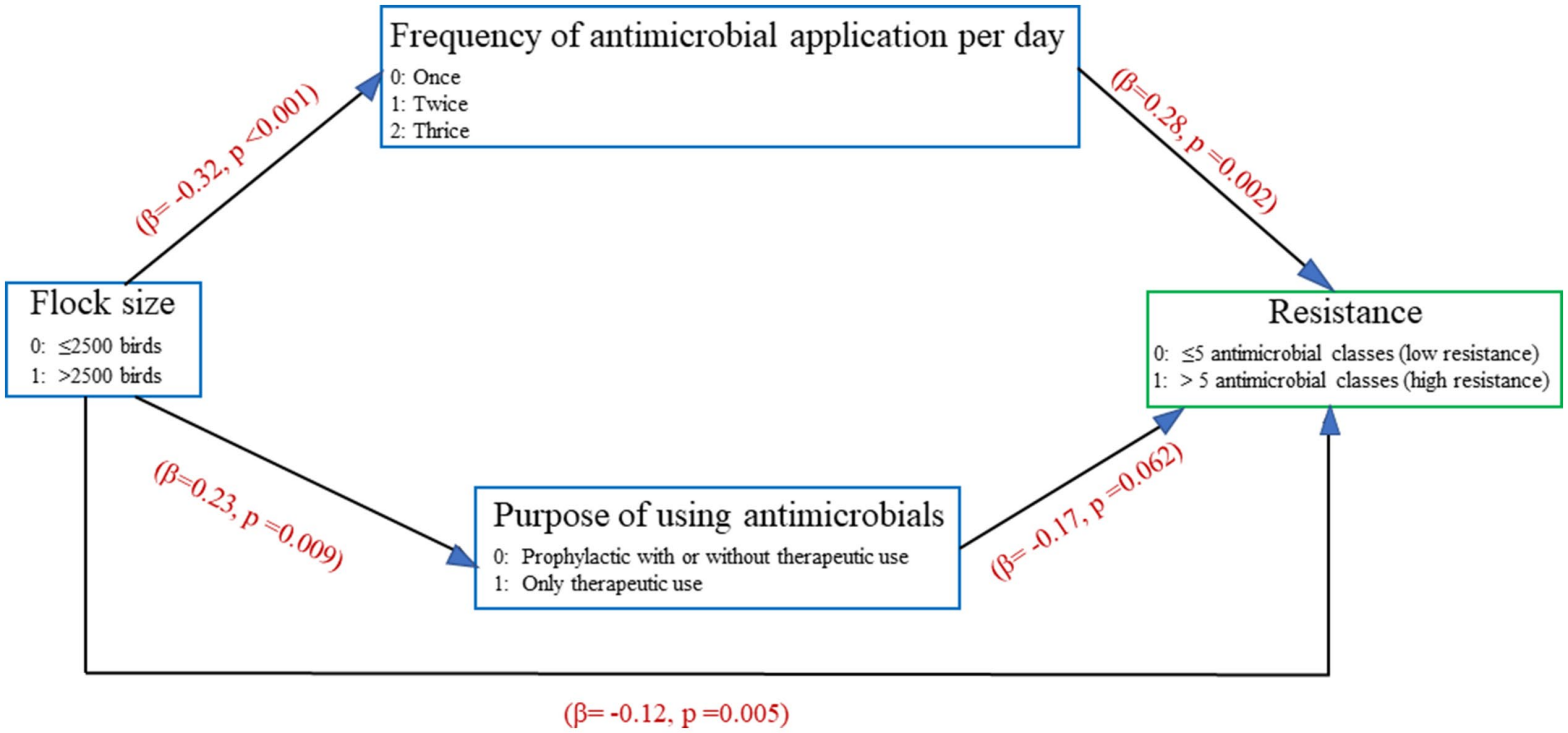
Final path analysis results for farm management factors that had an effect on increasing resistance on commercial chicken farms where *E. coli* was cultured.

The results of the path analysis (Figure 4) indicated that there was a “direct effect” of frequency of antimicrobial usage on higher resistance, with resistance increasing with more frequent administration of antimicrobials (*β* = 0.28, *p* = 0.002). Conversely, there was a “direct effect” of purpose of antimicrobial usage on lower resistance, with resistance marginally declining when antimicrobials were only administered therapeutically (*β* = −0.17, *p* = 0.062; or increasing when antimicrobials were administered prophylactically). There was an “indirect effect” of flock size on resistance, which decreased in larger flocks (*β* = −0.12, *p* = 0.005). Having larger flock sizes was associated with antimicrobial usage for therapeutic purposes only (*β* = 0.23, *p* = 0.009), while in smaller flocks antimicrobial usage for prophylactic purposes was more common (i.e. less antimicrobial usage for therapeutic purposes only *β* = −0.32, *p* < 0.001).

The other management factors were not significant at *p* < 0.05 in the final model. The model fitted the data well, as indicated by *χ*^2^ = 2.347 (*p* = 0.309), RMSEA = 0.040, CFI = 0.987 and SRMR = 0.042.

## 4 Discussion

The present study provides unique insights into the relationship between antimicrobial usage and resistance in *E. coli* and *Salmonella* spp. isolates cultured from commercial chicken farms in Bangladesh. It should be noted that isolates were cultured from cloacal swabs of apparently healthy birds and from their environment.

Commensal *E. coli* is present in chickens’ intestinal tracts, with a 100% recovery rate expected according to FAO guidelines (38). However, studies have reported differing recovery rates of *E. coli* from cloacal swabs from poultry, such as in Bangladesh (100–88%), Timor-Leste (85.5%), China (53.4%), Qatar (52.0%) and Pakistan (51.3%) (37, 39–43). In our study, we achieved a recovery rate of 73.8%. Varying recovery rates of *E. coli* between studies are likely influenced by transport and storage of samples, laboratory techniques used, and husbandry and management conditions on poultry farms (37).

In our study, *Salmonella* was detected in 27.3% of cloacal swabs. In other studies in Bangladesh, *Salmonella* spp. have been detected in 27–48% of cloacal swabs in poultry. Prevalence may differ due to differences in isolation and identification procedures, but also may be influenced by type of poultry, flock size and factors such as biosecurity, and hygiene (37, 40).

Some antimicrobials that were administered to chickens are classified as HPCIAs for human health, such as quinolones and polymyxins (33). Resistance to these antimicrobials was found on the studied farms. These antimicrobials are not recommended for use in animals without culture and antimicrobial susceptibility testing (34). Establishing a direct link between antimicrobial usage and resistance is challenging as multiple pathways exist and resistance might develop over many years. Nevertheless, this study identified that antimicrobial resistance patterns do not only depend on antimicrobial usage, as high levels of resistance were found on farms not reporting the use of these antimicrobials in the current production cycle. Furthermore, repeated administration of antimicrobials within a single day might have contributed to increased resistance. The impact of antimicrobial overuse on the development of antimicrobial resistance has been previously described (44) and selection pressure, cross-resistance and/or co-resistance might have contributed to the occurrence of antimicrobial resistance (32).

The presence of resistant bacteria in environmental samples is concerning as it represents a risk of contamination for areas surrounding poultry sheds. This contamination can occur through run off from poultry sheds, which may, in turn, contaminate water sources and the environment (45). Resistant bacteria can transfer resistance genes between different strains and across species (46). Such resistant commensal or pathogenic bacteria transfer can then result in resistant infections in humans, leading to treatment failure, higher medical costs, prolonged hospital stays, and increased mortality (47).

Antimicrobial agents may remain in the environment for years (48). Poor poultry husbandry practices, including inadequate disinfection, waste disposal into the environment, and poor infection control practices may contribute to the development of an environmental reservoir of resistant bacteria over time (49). The presence of resistant bacteria cultured from cloacal samples is also concerning because they may contaminate food products, resulting in foodborne illness (50).

Resistance was higher on small scale commercial chicken farms. This could be attributed to lower biosecurity standards and management practices, including inadequate housing facilities, or inadequate disinfection of water sources as reported by FAO (51). Our previous investigations into the biosecurity practices and antimicrobial usage on these farms revealed that inadequate biosecurity was more prevalent on farms with smaller flock sizes (<2,500 birds) compared to those with larger flocks (≥2,500 birds); and usage of antimicrobials was more common on small scale farms (6, 9). Small-scale farmers often lack formal training in biosecurity (13) and may be advised by representatives of pharmaceutical companies (8), and unqualified veterinary care givers who offer more affordable animal care (52). Although it is commonly reported that farmers are influenced by the advice of feed and chick traders (8), our study did not find evidence supporting this. Farmers rearing smaller flocks may more frequently administer antimicrobials prophylactically compared to farmers with larger flocks, to reduce the potential disease risk associated with poorer biosecurity.

Farms where antimicrobials were used prophylactically had higher levels of resistance in *E. coli*. Mass prophylactic administration of antimicrobials might result in lower adaptive immunity in chicken flocks (53), and their effectiveness in preventing poultry diseases may diminish over time (54), Thus, the misuse of antimicrobials in broiler (12) and layer (13) farms in Bangladesh may select for antimicrobial resistance in *E. coli* and potentially increase resistance.

We have used path analysis to investigate the factors contributing to high antimicrobial resistance. This approach enabled us to clarify complex interrelationships among variables, highlighting the most important pathways that were associated with the outcome (55). It allowed to display the direction and magnitude of both “direct” and “indirect” relationships between farm management factors and their impact on antimicrobial resistance in *E. coli*. However, although path analysis was used to analyze the hypothesized pathways, it is primarily based on correlations and cannot be used to prove causality (55).

The data collection in this study had several limitations. Firstly, while we could identify if an antimicrobial class was used during the current production cycle, we could not determine the frequency, dose or the duration of administration. Therefore, actual antimicrobial usage may have been underestimated. Secondly, some farmers might not have accurately remembered which antimicrobials they had administered. However, by focusing on antimicrobial use in the current production cycle, we aimed to minimize potential recall bias. Thirdly, antimicrobial susceptibility testing was performed using the disk diffusion method, which might not be optimal for detecting polymyxins (i.e. colistin in this study) resistance (25, 56). Colistin is a cationic, multicomponent, lipopeptide antimicrobial agent that diffuses slowly in antimicrobial susceptibility testing media, therefore the resulting zone of inhibitions tends to be inaccurate; and may bias the interpretation (57). Minimum inhibitory concentration (MIC) methods are preferred for colistin (25, 56), however, were not feasible to conduct in the laboratory in Bangladesh. In future studies, the use of MIC methods would be recommended for all antimicrobials, especially for colistin. However, MIC methods for colistin may also be affected by its cationic properties (58). Finally, the lack of veterinary breakpoints for most of the antimicrobials tested (25, 56) is a general limitation in veterinary studies, as breakpoints need to be extrapolated from humans or other species.

Overall, this study provides important baseline data that could be used to develop recommendations aimed at reducing antimicrobial usage in chicken production. Such recommendations may include initiatives for training farmers and raising awareness regarding the appropriate use of antimicrobials. Basic training in poultry pathology and necropsy would allow farmers to make informed decisions when selecting antimicrobials based on gross pathology. Routine availability of culture and antimicrobial susceptibility testing, and molecular techniques to identify and characterize commensal and pathogenic organisms, along with residue testing performed by governmental authorities, could also form integral components of an effective antimicrobial stewardship program for Bangladesh.

## Data Availability

The raw data supporting the conclusions of this article will be made available by the authors, without undue reservation.
