# Supplementary material for: Association between antimicrobial usage and resistance on commercial broiler and layer farms in Bangladesh
Source: Front Vet Sci. 2024 Aug 29;11:1435111. doi: 10.3389/fvets.2024.1435111 (PMC11390387; doi:10.3389/fvets.2024.1435111)
Supplement: Supplementary file 1 [file Data_Sheet_1.pdf]

Supplementary material for paper “Association between antimicrobial usage and resistance on commercial broiler and layer farms in Bangladesh”

Bacterial culture

Escherichia coli

The sample (10 µl) was streaked onto a MacConkey (MC) agar plate (Neogen corporation) incubated aerobically overnight at 37°C. Any suspected *E. coli* colonies were subcultured onto Eosin methylene blue (EMB) agar (Neogen corporation) and incubated aerobically at 37ºC for 18-24 hours. Suspected colonies (MC agar, lactose positive; EMB, metallic green sheen) were subcultured onto 5% blood agar (Blood agar base, (BA) (Neogen corporation). If biochemical results were Triple Sugar Iron Agar, fermentation of glucose and gas production but no sulphur reduction; indole reaction, positive; citrate utilisation, negative then the sample was considered as *E. coli* positive. All positive isolates were stored at -80°C in brain heart infusion broth with 50% glycerol (Neogen corporation) (Kakkar et al., 2018).

***Salmonella* spp.**

The sample, (100 µl overnight culture, divided into 3 separate drops was plated on to novobiocin (Oxoid Ltd., Basingstoke, Hampshire, RG24 8PW, UK) supplemented Modified Semisolid Rappaport Vassiliadis (MSRV) agar (Oxoid Ltd.) and incubated for 24 hours at 41.5°C. Production of any gray white, turbid zone from the centre of the inoculation on the MSRV agar plates was suspected *Salmonella* and was streaked onto brilliant green (BG) agar (Oxoid Ltd.) and xylose lysine deoxycholate (XLD) agar (Neogen corporation) by using an inoculating loop which was dipped into the periphery of the opaque zone. Plates were incubated overnight aerobically at 37°C. After incubation the plates were examined for the presence of typical colonies of *Salmonella*. In case of BGA, light pink colony against a rose-pink background and red colonies with black centers on XLD agar were observed. Any suspected *Salmonella* colonies on BG or XLD agar were verified and confirmed by biochemical tests

including Triple Sugar Iron agar slant reaction (dextrose fermentation, H2S produced), indole reaction (negative) and citrate utilisation test (positive).

Suspected *Salmonella* colonies were transferred to 5% blood agar (Blood agar base, Oxoid Ltd.) incubated overnight aerobically at 37°C and stored at -80°C in brain heart infusion broth with 50% glycerol (Neogen corporation).

Antimicrobial susceptibility testing

E. coli and *Salmonella* spp. underwent Kirby-Bauer disk diffusion antimicrobial susceptibility testing against 12 antimicrobials: amoxicillin (10 µg), ampicillin (10 µg), cephalexin (30 µg), doxycycline (30 µg), erythromycin (15 µg), enrofloxacin (5 µg), gentamicin (10 µg), neomycin (10 µg), ciprofloxacin (5 µg), azithromycin (15 µg), colistin (10 µg), pefloxacin (5 µg), sulphonamide and trimethoprim (25 µg). These antimicrobials belong to the following classes:

Beta lactams	Amoxicillin
	Ampicillin
	Cephalexin
Tetracycline	Doxycycline
Macrolide	Erythromycin
	Azithromycin
Quinolone	Enrofloxacin
	Ciprofloxacin
	Pefloxacin
Polymyxin	Colistin
Sulphonamides	Sulphonamide and trimethoprim
Aminoglycosides	Gentamicin
	Neomycin

E. coli and *Salmonella* colonies from BA were mixed with phosphate buffer saline (PBS) by vortexing and the turbidity adjusted to the MacFarland 0.5 turbidity standard. The broth was streaked on to a Mueller Hinton agar plate (Difco Laboratories, Sparks, MD, USA). Antibiotic discs (HiMedia (Vadhani Industrial Estate, L.B.S. Marg, Mumbai - 400 086, India) for *E. coli* and Oxoid (Thermo Fisher Scientific, 168 Third Avenue, Waltham, MA USA 02451) for *Salmonella* spp. were applied aseptically onto the surface of the inoculated plates with multidisc dispenser.

The animal CLSI guidelines were used to interpret enrofloxacin (5 µg) as it is the only antimicrobial which has a veterinary breakpoint for poultry (CLSI, 2019). The human CLSI guidelines were used to interpret amoxicillin (30 µg), ampicillin (25 µg), cephalexin (25 µg), doxycycline (30 µg), erythromycin (15 µg), gentamicin (10 µg), neomycin (30 µg), azithromycin (30 µg), pefloxacin (5 µg), and trimethoprim sulphonamides (25 µg) (CLSI, 2018). For colistin (10 µg), the breakpoint used was what was recommended by the manufacturer (Rosco Diagnostica, Taadtrugaardsvej 30, 2630 Denmark). *E. coli* ATCC25922, and *Salmonella* B41 (S. Kentucky)/2010/Bangladesh, were used for quality control (QC) and susceptibility testing. .

Table S1. Results of antimicrobial susceptibility testing by antimicrobial classes of *E. coli* and *Salmonella* spp. isolates cultured from cloacal and environmental samples collected on commercial chicken farms in the Chattogram district of Bangladesh

**Antimicrobial classes**	***E. coli*** **Resistance % (N farms)**			***Salmonella* spp. Resistance % (N farms)**		
	**Overall (N=107)**	**Cloacal (N=79)**	**Environmental (N=81)**	**Overall (N=11)**	**Cloacal (N=3)**	**Environmental (N=10)**
Amoxicillin	100.0 (107)	100.0 (79)	100.0 (81)	100.0 (11)	100.0 (3)	100.0 (10)
Ampicillin	100.0 (107)	100.0 (79)	100.0 (81)	100.0 (11)	100.0 (3)	100.0 (10)
Cephalexin	100.0 (107)	100.0 (79)	100.0 (81)	100.0 (11)	100.0 (3)	100.0 (10)
Doxycycline	100.0 (107)	100.0 (79)	100.0 (81)	90.9 (10)	100.0 (3)	90.0 (9)
Erythromycin	100.0 (107)	98.7% (78)	98.8 (80)	Not performed		
Azithromycin	92.5 (99)	89.9 (71)	91.4 (74)			
Enrofloxacin	95.3 (102)	92.4 (73)	86.4 (70)	100.0 (11)	100.0 (3)	100.0 (10)
Ciprofloxacin	Not performed			100.0 (11)	100.0 (3)	100.0 (10)
Pefloxacin	93.5 (100)	91.1 (72)	85.2 (69)	100.0 (11)	100.0 (3)	100.0 (10)
Colistin	5.6 (6)	3.8 (3)	3.7 (3)	18.2 (2)	0.0 (0)	20.0 (2)
Sulphonamide and trimethoprim	100.0 (107)	97.5 (77)	95.1 (77)	54.5 (6)	100.0 (3)	30.0 (3)
Gentamicin	83.2 (89)	82.3 (65)	74.1 (60)	72.7 (8)	33.3 (1)	70.0 (7)
Neomycin	75.7 (81)	72.2 (57)	67.9 (55)	81.8 (9)	33.3 (1)	80.0 (8)

Resistant includes isolates identified with resistant and intermediate susceptibility.

Table S2. Antimicrobial classes used on commercial layer and broiler farms where *E. coli* (N=107) and *Salmonella* spp. (N=11) was cultured in Chattogram, Bangladesh.

**Antimicrobial classes**	**Usage % (N farms) on farms where *E. coli* was cultured**	**Usage % (N farms) where *Salmonella* spp. was cultured**
Quinolones	56.1 (60)	45.5 (5)
Polymyxins	43.0 (46)	63.6 (7)
Macrolides	30.8 (33)	45.5 (5)
Aminoglycosides	29.9 (32)	27.3 (3)
Beta lactams	30.8 (33)	54.5 (6)
Sulphonamides	29.0 (31)	18.2 (2)
Tetracyclines	50.5 (54)	63.6 (7)

Table S3. Antimicrobial classes not used on commercial layer and broiler farms where *E. coli* (N=107) was cultured in Chattogram, Bangladesh and resistance to these antimicrobial classes.

**Antimicrobial classes**	**N farms where *E. coli* was cultured, but antimicrobial classes were not used**	**Resistance % of non-user farms (N non-user farms resistant/Total N non-user) where *E. coli* was cultured**
Quinolones	47	95.7 (45/47)
Polymyxins	61	8.2 (5/61)
Macrolides	74	100.0 (74/74)
Aminoglycosides	75	81.3 (61/75)
Beta Lactams	74	100.0 (74/74)
Sulphonamides	76	100.0 (76/76)
Tetracyclines	53	100.0 (53/53)

References

CLSI. (2018). *Performance standards for antimicrobial susceptibility testing, 28th edition*. https://clsi.org/media/1930/m100ed28_sample.pdf

CLSI. (2019). *Performance standards for antimicrobial suceptibility testing*

Kakkar, M., Chatterjee, P., Chauhan, A. S., Grace, D., Lindahl, J., Beeche, A., Jing, F., & Chotinan, S. (2018). Antimicrobial resistance in South East Asia: time to ask the right questions. *Glob Health Action, 11*(1), 1483637. https://doi.org/10.1080/16549716.2018.1483637
